# Supplementary material for: A Highly Sensitive Chitosan-Based SERS Sensor for the Trace Detection of a Model Cationic Dye
Source: Int J Mol Sci. 2024 Aug 28;25(17):9327. doi: 10.3390/ijms25179327 (PMC11395516; doi:10.3390/ijms25179327)
Supplement: Supplementary file 1 [file ijms-25-09327-s001.zip › ijms-3163738-supplementary.pdf]

SUPPLEMENTARY DATA

A highly sensitive chitosan-based SERS sensor and its application for trace detection of a model cationic dye

Bahareh Vafakish and Lee D. Wilson *

Department of Chemistry, University of Saskatchewan, 110 Science Place, Thorvaldson Building, Saskatoon, SK S7N 5C9, Canada; baharheh.vafakish@usask.ca

* Correspondence: lee.wilson@usask.ca; Tel.: +1-306-966-2961

Table of Contents

Scheme S1: Synthetic route of (a) CS-SAMSA and (b) Ag@CS-SAMSA 11

Figure S1: Extrapolation to obtain the limiting viscosity number of Mark-Houwink-Sakura equation between intrinsic viscosity of CS-SAMSA solutions and their concentrations 12

Figure S2: FT-IR spectra of mercapto succinic acid and S-Acetyl mercapto succinic anhydride (SAMSA) 13

Figure S3: FT-Raman spectra of CS and CS-SAMSA 14

Figure S4: FT-Raman spectra of CS-SAMSA (static scan) centered at 2550 cm^{-1} 15

Figure S5: FT-NIR spectra of CS and CS-SAMSA 16

Figure S6: Differential Thermal Gravimetry (DTG) profile of CS and CS-SAMSA 17

Figure S7: XPS (a) wide scan and (b) high resolution spectra (c) deconvoluted peaks.............................19

Figure S8: Different appearance of Ag@CS-SAMSA (right) and Ag@CS (left) solution 20

Figure S9: (a) FT-Raman Spectra of CS (black) and Ag@CS (purple). The highlighted region shows the main difference after the addition of Ag NP. (b) FT-Raman spectra of CS (black) and Ag@CS (purple), static scan centered at 2550 cm^{-1}...21

Figure S10: UV-Vis adsorption spectra of Ag@CS-SAMSA which shows the LSPR peak at 720 nm, close enough to the laser excitation wavelength of 785 nm...22

Figure S11: The black line is the subtracted Raman spectrum of Solid MB (black) and MB (10 µL, 100 µM) on Ag@CS-SAMSA (Red)...23

Figure S12: SERS spectra of MB (10 µL, 10 µM) on Ag@CS..24

Figure S13: Raman spectrum of Ag@CS-SAMSA with MB (10 µL, 10 µL) Static scan centered at 1396 cm-1 (SERS peak)...25

Figure S14: Top: Focus of SERS peak centered at 1396 cm-1 Bottom: calibration curve to calculate LOD and LOQ 26

Figure S15: Adsorption isotherm of MB on SERS substrate fitted with (a) Langmuir and (b) Sips model. MB concentration: 1 nm to 100 µM, contact time 1 h at room temperature..27

Figure S16: Schematic representation of adsorbed MB on Ag@CS-SAMSA to represent the binding geometry of MB with the substrate...28

Figure S17: The Stokes Raman shift of the SERS band for substrate with MB (Red) and solid MB (Black). MB concentration (10 µM, 10 µL) 29

Figure S18: Schematic representation of adsorbed MB on Ag@CS-SAMSA to show the distance between MB and Ag NP..30

Figure S19: Effect of Ag NP concentration in the CS-SAMSA substrate. The concentration of $AgNO_3$ solution for 50 mg Ag/g of substrate is 50mM, and for 1500 mg Ag/g of substrate is 150mM.................31

For SERS studies MB (10 µM, 10 µL) was dispersed on different substrate and dried at room temperature for 1 hour. 31

Figure S20: Regeneration cycles for Ag@CS-SAMSA loaded with MB. Adsorption cycle condition: MB concentration (10 µL, 10 µM) Desorption cycle condition: methanol and water washing........................32

Figure S21: Reproducibility of the SERS substrate by MB (10 µL, 10 µM) for 2 selected sites of five different batches. The intensities are: 3.1, 3.2, 2.9, 3.0, 3.1, 3.4, 2.9, 3.1, 3.0, 2.9 (×103 cps) and the RSD% is 5.2% 33

Figure S22: Storage Stability of substrate after 30days tested on 0, 5, 10, 20 and 30days and the Raman scattering intensity was compared. MB (10 µL, 10 µM) 34

Table S1: The spectral positions and assignments of the Raman signatures 35

Table S2: Langmuir and Sips best-fit adsorption parameters for MB with Ag@CS-SAMSA 36

Table S3: EF and LOD of similar reported substrates in the literature in compare to the results of this study 37

LOD and LOQ Calculation .... **Error! Bookmark not defined.**

Enhancement Factor (EF) Calculations .... **Error! Bookmark not defined.**

S1. Methods

S1.1. Synthesis of S-Acetyl mercaptosuccinic anhydride (SAMSA)

A synthetic method was adapted herein with some modification (Klotz & Heiney, 1962). In brief, a suspension of mercaptosuccinic acid (1 g, 6.7 mmol) in acetyl chloride (2 mL, 28.0 mmol) was heated on an oil bath and refluxed for 2 h. The obtained clear pale-yellow solution was cooled in an ice bath to afford SAMSA as a white powder which was washed with cold diethyl ether on Buchner filter and dried in vacuum desiccator overnight.

S1.2. Synthesis of CS-SAMSA

Low-molecular-weight chitosan (2.4 g) was suspended in 250 mL water. The mixture was stirred for 30 minutes at room temperature, followed by the addition of pyridine (50 mL) to reach pH=7.5 with stirring for another 30 minutes. SAMSA (1.5 g, 1.03 mmol) was added to the mixture. The reaction mixture was stirred for 48 h at room temperature. Then, the mixture was concentrated under vacuum to afford an off-white powder that was washed in a Soxhlet extractor with acetone overnight, followed by drying in an

oven at 50°C for 6 h. The dried powder was subsequently suspended in 50 ml of phosphate buffer when hydroxylamine (1.7 g, 51 mmol) was added in small portions. The mixture was stirred at room temperature for 2 h. The yellowish precipitate was filtered off and washed with water, followed by drying in the oven at 50 °C for 6 h.

S1.3. Synthesis of Ag@CS-SAMSA

$AgNO_3$ solution (15 mM, 4 ml) was cooled in an ice bath for 15 min. Subsequently, $NaBH_4$ solution (0.04 M, 200 µL) was added dropwise with vigorous stirring. Then, the mixture was stirred continuously in the ice bath for 30 min after complete addition of $NaBH_4$, similar to previous reports with slight modification (Dolatkhah & Wilson, 2016), CS-SAMSA or CS (45 mg) was dispersed in the solution and mixed for more 30 min followed by sonication for 10 min and then allowed to settle at room temperature overnight. The dispersion was centrifuged, and the obtained powder was dried in an oven at 50°C for 6 h. A variable concentration $AgNO_3$ solution was used to prepare Ag@CS-SAMSA (cf. Fig. S18).

S2. Preparation of SERS Substrate

A fine powder of Ag@CS-SAMSA (0.05 g) was re-dispersed in Millipore water (1 mL) with the aid of sonication and then drop-casted onto a regular microscope glass slide (4 cm^2) to form a uniform film which was oven dried at 50°C for 6 h.

S3. Materials Characterization

S3.1 Viscometry

The viscosity-average molecular weight of CS-SAMSA was determined by intrinsic viscosity (Xue & Wilson, 2022). CS-SAMSA was dissolved in 0.1 M acetic acid/0.2 M sodium chloride to make solutions with variable concentrations (1.0 to 5.0 g/L). After dissolution, each sample was filtered through Whatman no. 4.0 filter paper.

The viscosity of each sample was measured at 25 ± 0.5 °C using Ubbelohde capillary viscometer in a water bath. The intrinsic viscosity of each sample was calculated by Eqn. S1 (Chattopadhyay & Inamdar, 2010; Costa, Teixeira, Delpech, Souza, & Costa, 2015);

$$\eta_i = \frac{(t_n - t_0)/t_0}{C} \tag{S1}$$

where t_n is the flow time of each CS-SAMSA solution, t_0 is the flow time for solvent and η_i is the intrinsic viscosity of the according solution. Upon extrapolation of the viscosity of the solutions versus their concentration, the limiting intrinsic viscosity was found. The viscosity-average molecular weight (MWt) is calculated by using Mark-Houwink-Sakurada equation (S2);

$$[\eta_i] = K\,[M_v]^{\alpha} \tag{S2}$$

where Mv is the viscosity-average MWt of CS-SAMSA, α and K are constants ($K = 1.81 \times 10^{-6}$ L/g and $\alpha = 0.93$) for the solvent system employed (De Oliveira, Franco, & Oliveira Junior, 2014).

S3.2 Infrared (IR) Spectroscopy

FT-IR spectra were recorded using a Bio-RAD FTS-40 spectrophotometer in reflectance mode. All samples were ground into fine powders and completely mixed with spectroscopic grade KBr with a fixed ratio 1 to 10 (w/w). The DRIFT spectra were recorded between 400 and 4000 cm^{-1} (Kubelka-Munk intensity units) with a resolution of 4 cm^{-1} and multiple scans (n=32) that were corrected relative to a background reference spectrum of KBr. The absorbance mode was used following the Kubelka-Munk method, which is effective for studying solid samples and interpreting spectral data.

S3.3 ^{13}C Solids NMR Spectroscopy

^{13}C solids NMR spectra were obtained with a Bruker AVANCE IIIHD spectrometer using a 4 mm DOTY CP-TOSS (Total Sideband Suppression) with a fixed spinning speed (7.5 kHz) operating at 125.77 MHz while the ^{1}H-NMR frequency was 500 MHz. The proton 90° pulse of 5 μs was applied with the contact time of 1 ms and the ramp pulse on the proton channel. The number of scans was 2000-4000 with a recycle delay of 2 s. All spectra were externally referenced to adamantane at 38.48 ppm. During the acquisition, the SPINAL-64 ^{1}H decoupling sequence was applied with a frequency of 50 kHz.

S3.3 Thermogravimetry (TG) Analysis

To study the sample thermal gravimetry (TG) profile, a TA instrument Q50 TGA system working with a heating rate of 5°C min^{-1} up to 500 °C was employed with an aluminum sample pan. Nitrogen served

as the carrier gas with a flow rate of 90 mL/min. The results were plotted as the first derivative of the TG profile with temperature (% °C^{-1}; DTG) against temperature (°C).

S3.4 Raman Spectroscopy

The optical absorbance spectrum between 200-800 nm was recorded by a Varian Cary 100 scan spectrophotometer equipped with a solid sample accessory in transmission mode. A Renishaw InVia reflex Raman microscope with a Pelletier cooled CCD (charged coupled device) detector was employed for generation of one-dimensional (1D) Raman sample spectra. The excitation source was a solid-state diode laser at 785 nm with a 1200 lines/mm grating system. The instrument wavelength was calibrated at 520 cm^{-1} using an internal Si (110) sample. The laser was focused with a 20× focal-lens objective (numerical aperture = 0.40). For the case of high resolution two dimensional (2D) Raman imaging, the same instrument described above for the 1-D Raman spectral profile with a 50× focal-lens objective (numerical aperture = 0.50). A similar procedure from a previously reported study was used with slight modification (Xue & Wilson, 2019b). The streamline mode was set in the instrument software (Renishaw Wire V3.4). The exposure time was 12 s centered at 225 or 1396 cm^{-1}. The baseline linearization and normalization were performed using the software before the creation of images to minimize any artefacts. The color intensity of each pixel in the image was attributed to the integration of the corresponding signature (225 and 1396 cm^{-1}).

S3.5 Near Infrared (NIR) Spectroscopy

FT-NIR spectra were recorded with a Bruker MPA II multipurpose FT-NIR in the range of 3000-1000 nm with 4 nm interval in diffuse reflection mode using an integrating sphere accessory for solid samples. Multiple scans (n = 16) were collected for greater signal-to-noise ratios.

S3.5 Transmission electron microscopy (TEM)

TEM images were collected by a Hitachi HT-7700 microscope at voltage of 100 kV. The fine powder of the sample was deposited onto a carbon-coated TEM grid.

S3.6 X-ray Photoelectron Spectroscopy (XPS)

XPS measurements were obtained using a Kratos (Manchester, UK) AXIS Supra system. The source was a 500 mm Rowland circle monochromator Al K-α (1486.6 eV) source, equipped with a hemi-spherical (HAS) and a spherical mirror analyzer (SMA). The size of hybrid slot was 300×700 microns. The range of binding energy used was -5 to 1200 eV with 1 eV step and a pass energy of 160 eV. To acquire the high-resolution scans, 0.05 eV steps with a pass energy of 20 eV were applied. An emission current of 15 mA and an accelerating voltage of 15 keV was used for data collection. All the binding energies were calibrated to C*1s* (280.4 eV).

S3.7 Surface Enhanced Raman Spectroscopy (SERS)

Before MB detection, the Raman spectra of the SERS substrate was recorded, serial dilutions of MB (*aq*) solutions from 1 nM to 100 μM were prepared. 10 μL aliquots stock MB solutions at variable concentrations were drop casted onto the previously prepared substrate, where it was kept at 23°C for 1 h to allow for complete dryness. The SERS substrate containing MB was exposed to the excitation laser to acquire 1D Raman spectra. Calibration curves were obtained to enable estimation of LOD and LOQ, which employed variable dye concentrations of MB solution onto a SERS substrate. To assess the effect of Ag NPs concentration in the SERS substrate, a fixed concentration of MB (10 μM) was used. The same strategy was applied to Ag@CS substrate. To investigate the reusability of the substrate, an Ag@CS-SAMSA substrate was modified with the MB dye probe molecule (10 μL, 10 μM) washed with plenty amount of methanol followed by washing with DI water (5 times) and dried at 50°C overnight. The washed substrate was studied for regeneration up to three cycles. The reproducibility of the substrate was evaluated by the same concentration of the dye probe molecule with the various individually fabricated substrates in five independently prepared batches. To study the long-term storage stability of the substrate, the MB solution (10 μL, 10 μM) was dropped onto the Ag@CS-SAMSA substrate, which were kept at 23°C. The Raman scattering spectra were recorded at variable times (0, 5, 10, 20 and 30 days).

S4. Sample Calculations

LOD and LOQ Calculations

The limit of detection (LOD) was calculated by the following equation

LOD = $3\sigma/s$

Here σ is the standard deviation of the SERS intensity signals at 1396 cm^{-1}, and s is the slope of the calibration curve (Figure S12), hence:

LOD = (3× 0.0053) / 1.02 = 1.6 nM = 0.54 µg/L

The limit of quantitation (LOQ) was calculated by the following equation

LOQ = $10\sigma/s$

Here σ is the standard deviation of the SERS intensity signals at 1396 cm^{-1}, and s is the slope of the calibration curve (Figure S12), hence:

LOQ = (10× 0.0053) / 1.02 = 1.6 nM = 5.4 nM = 1.7 µg/L

Enhancement Factor (EF) Calculations

The EF is calculated by:

$$EF = \frac{I_{SERS}}{I_{bulk}} \times \frac{N_{bulk}}{N_{SERS}}$$

The ratio of $\frac{N_{bulk}}{N_{SERS}}$ is calculated by:

$$\frac{N_{bulk}}{N_{SERS}} = \frac{2344\,\lambda}{NA^2} \times A \times \frac{\rho}{w}$$

32 mg MB was dissolved in 10 ml Millipore water and diluted to make a 10 mM solution. 100 µL diluted to 10 ml to make a 100 µM solution. A 10 µM solution was made by 10 times dilution of 100 µM solution. 10 µL (0.1 nmol of MB) was spread on a substrate area of 4 cm^2. Density of MB crystal is 1.76 g cm^{-3} (Based on IARC monograph-108 for Methylene Blue)

λ = 785 nm

Numerical aperture (N.A.) = 0.4 (for ×20 lens)

A = 4 cm^2

ρ = 1.76 gcm^{-3}

W = 32 ng

$$\frac{Nbulk}{NSERS} = \frac{2344\ (785)}{(0.4)^2} \times 4 \times \frac{1.76}{32} = 2.5 \times 10^6$$

$$\frac{I_{SERS}}{I_{bulk}} = 105$$

EF = 2.6×10^8

S5. Supplementary Schemes and Figures

(a)

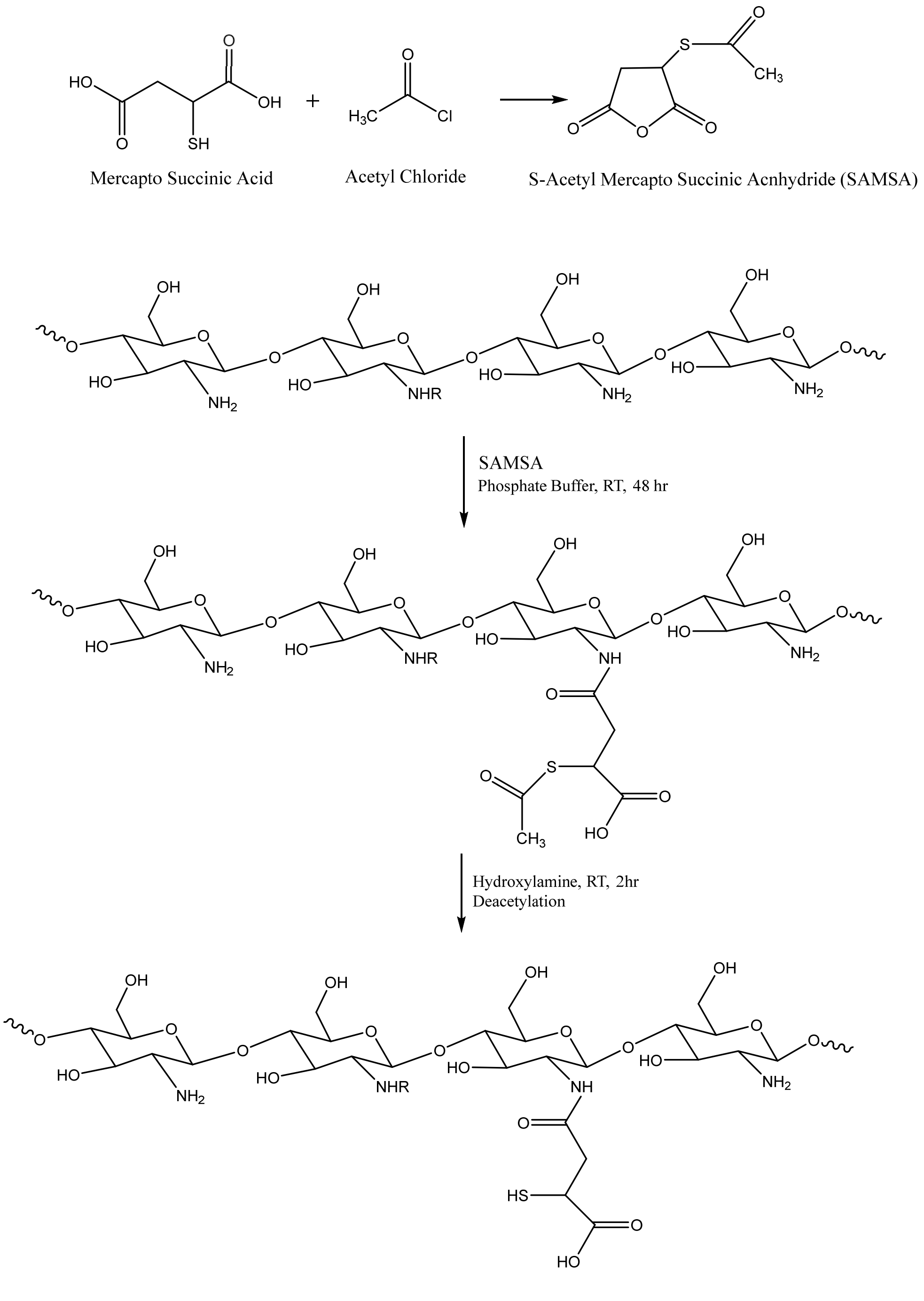

CS-SAMSA

(b)

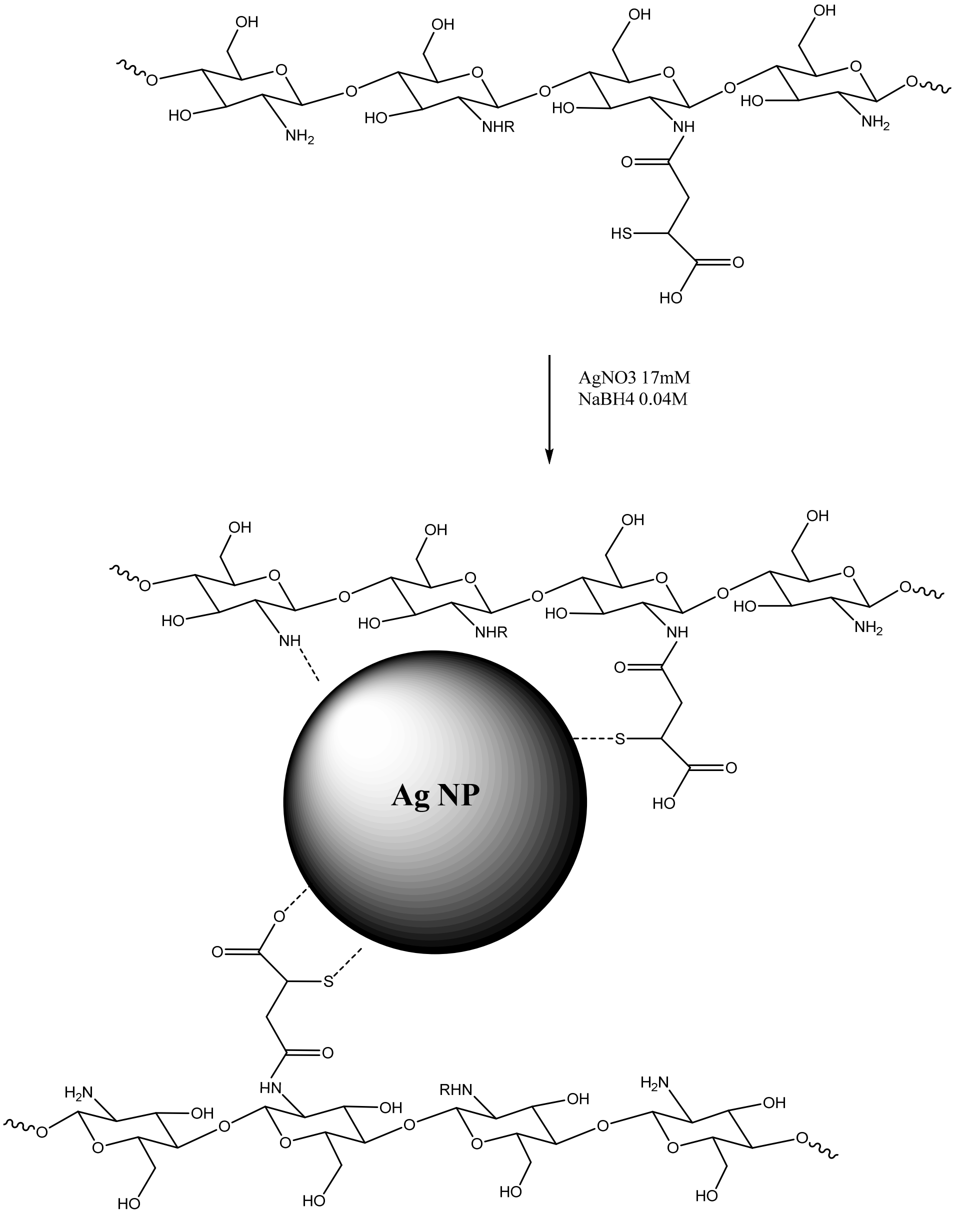

Ag@CS-SAMSA

Scheme S1: Synthetic route of (a) CS-SAMSA and (b) Ag@CS-SAMSA.

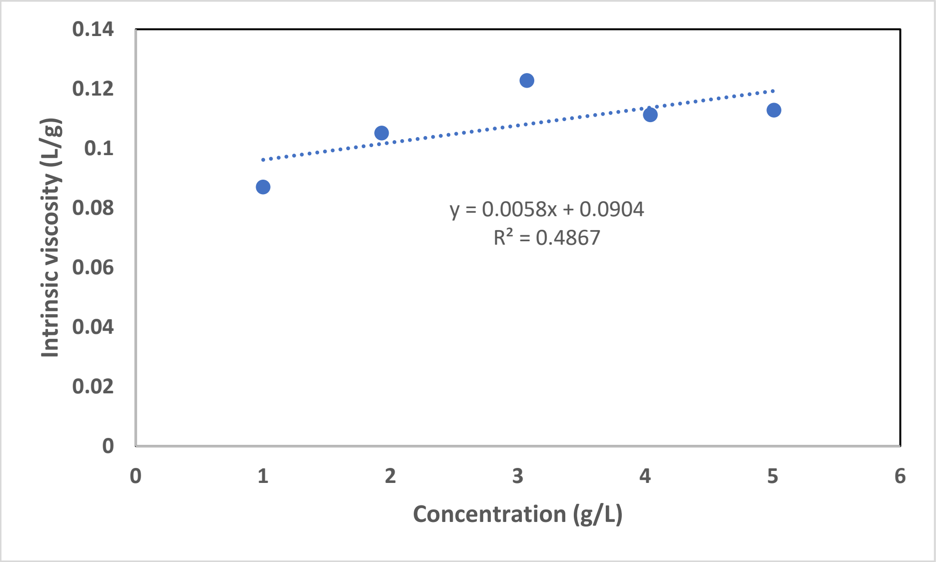

Figure S1: Extrapolation to obtain the limiting viscosity number of Mark-Houwink-Sakurada equation between intrinsic viscosity of CS-SAMSA solutions and their concentration.

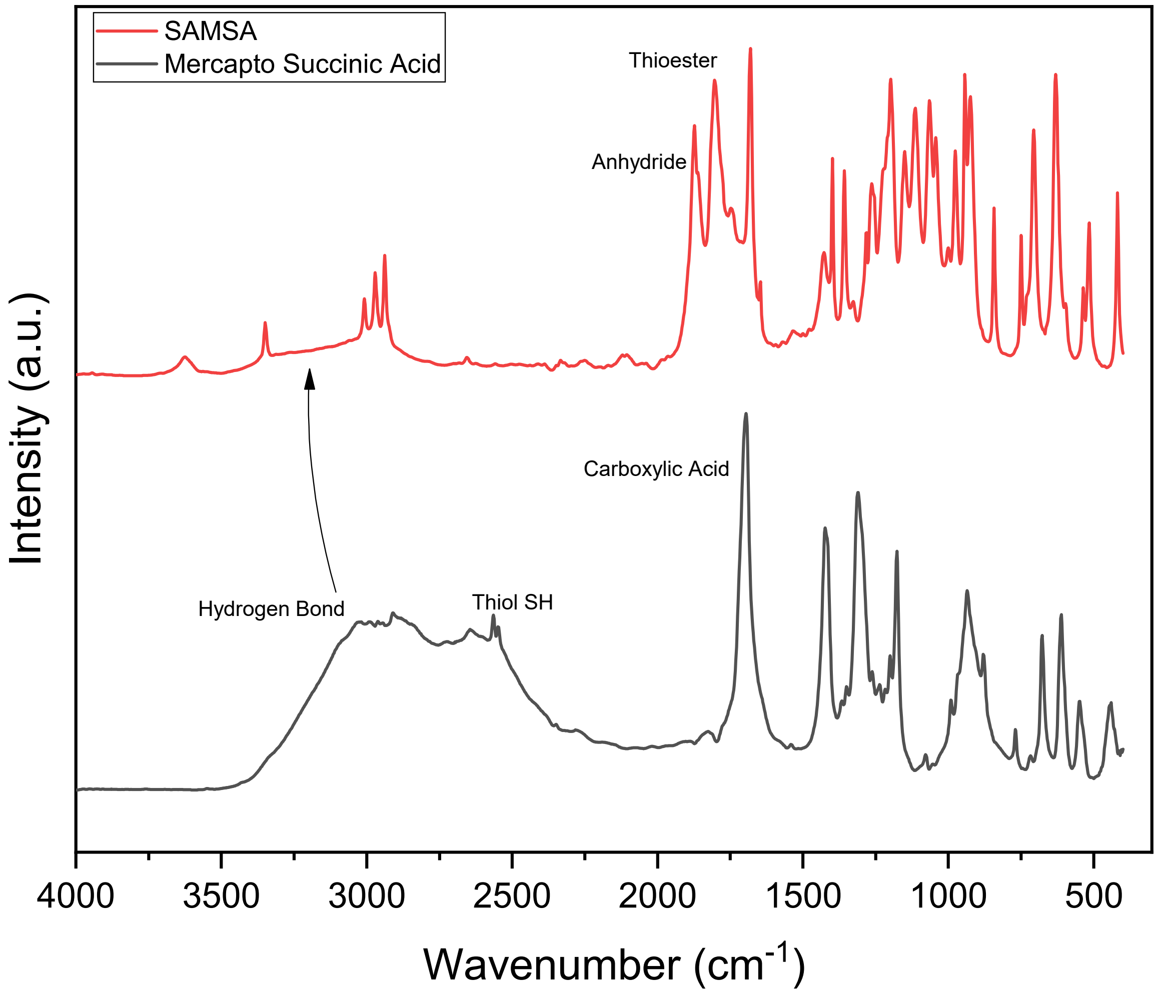

Figure S2: FT-IR spectra of mercapto-succinic acid and S-acetyl mercapto-succinic anhydride (SAMSA).

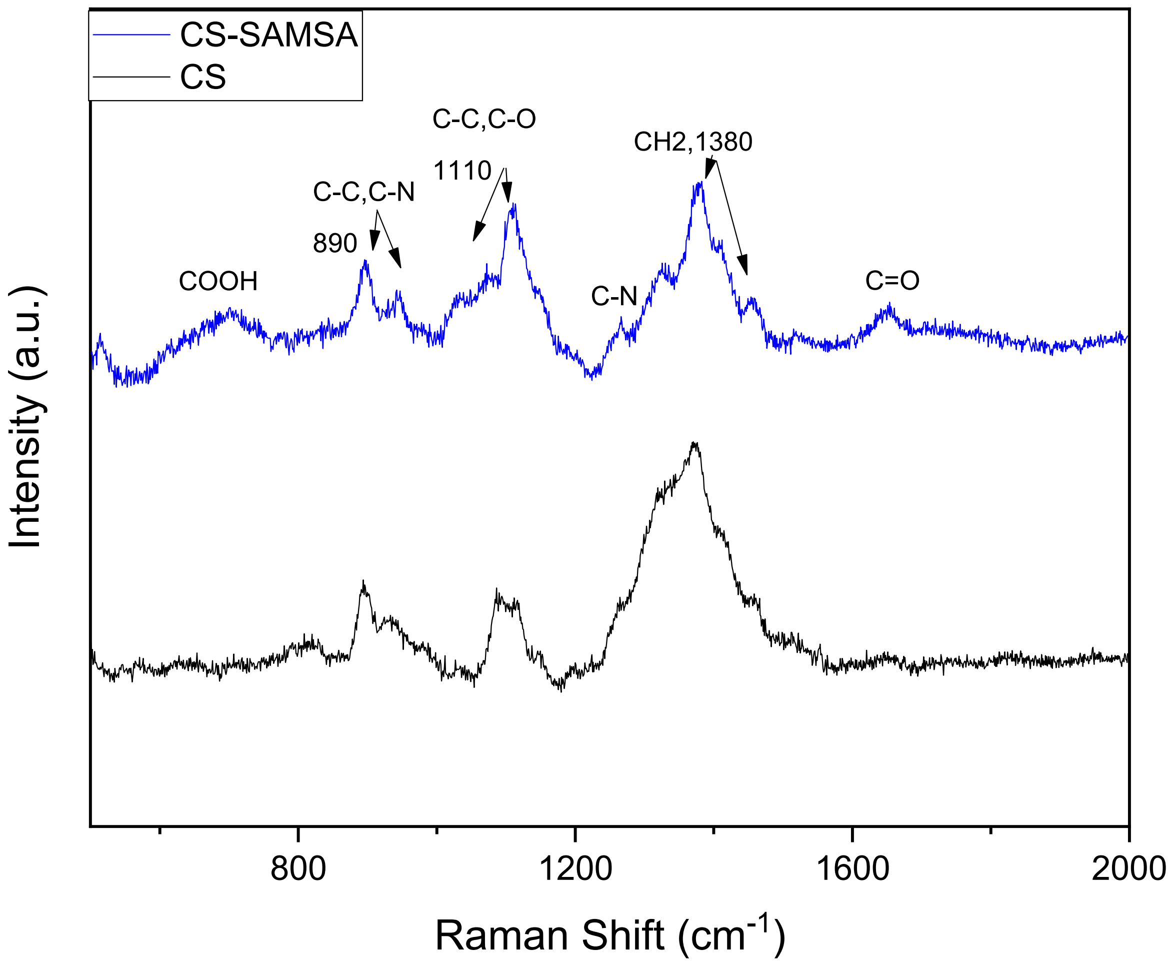

Figure S3: FT-Raman spectra of CS and CS-SAMSA.

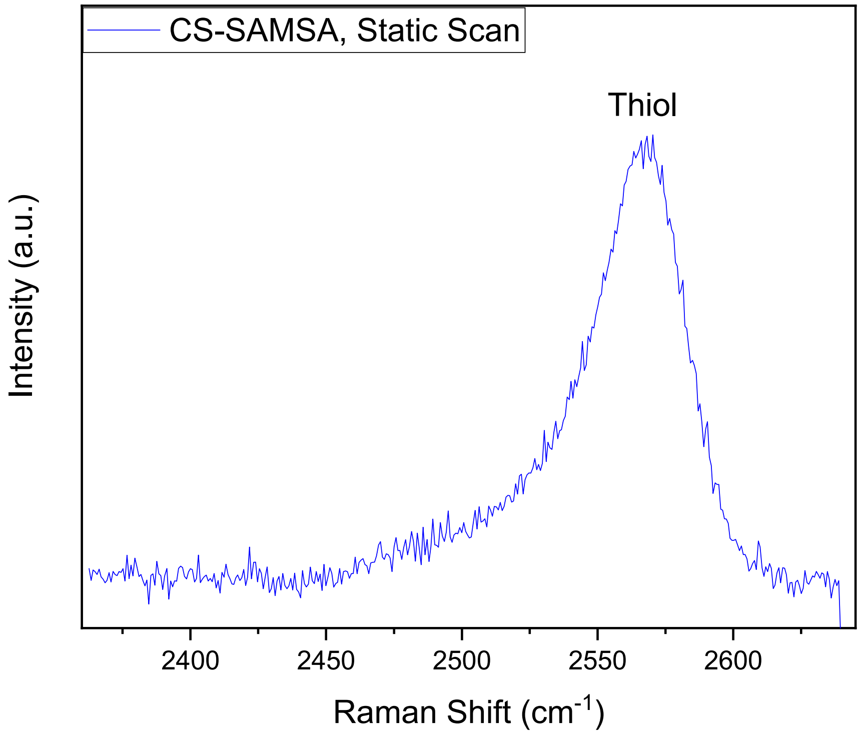

Figure S4: FT-Raman spectra of CS-SAMSA (static scan) centered at 2550 cm^{-1}.

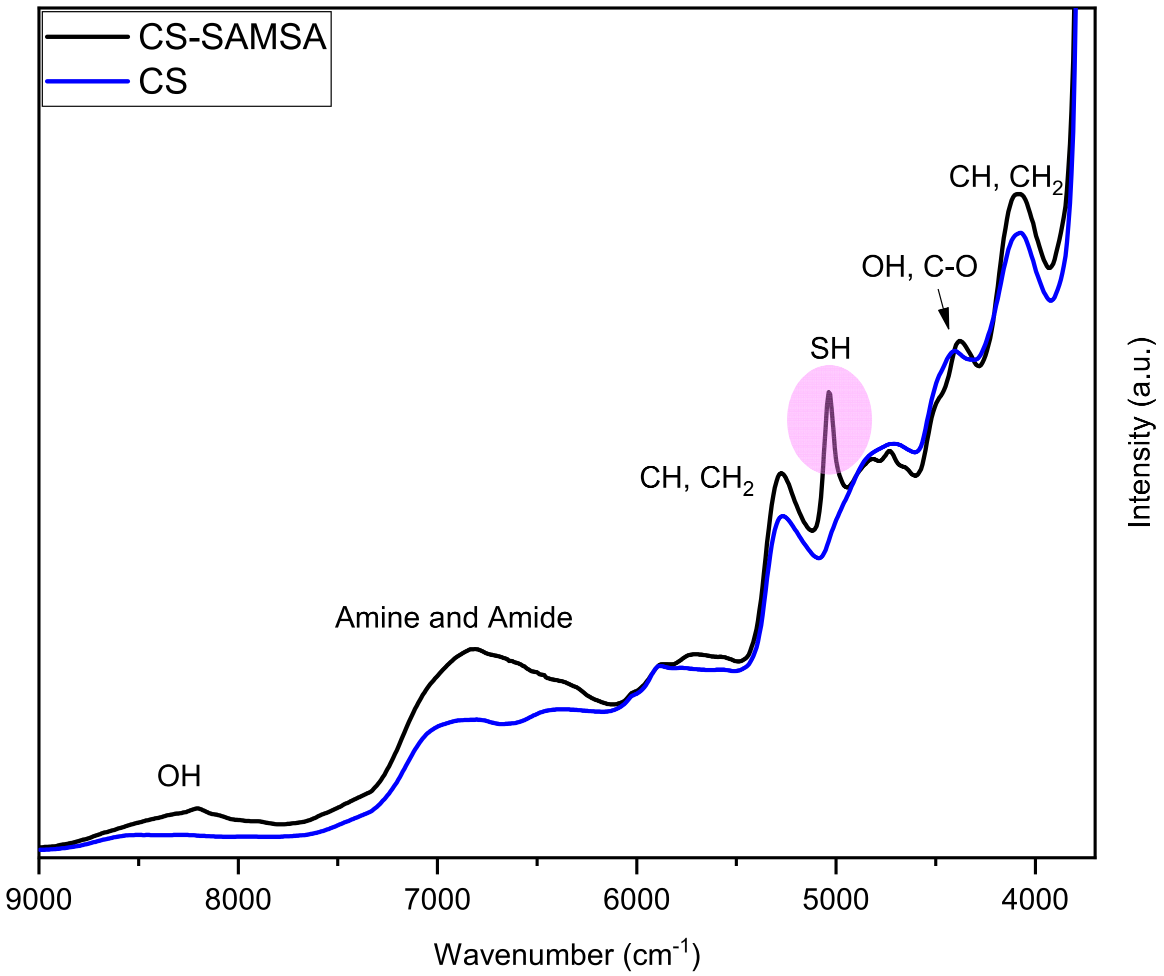

Figure S5: FT-NIR spectra of CS and CS-SAMSA.

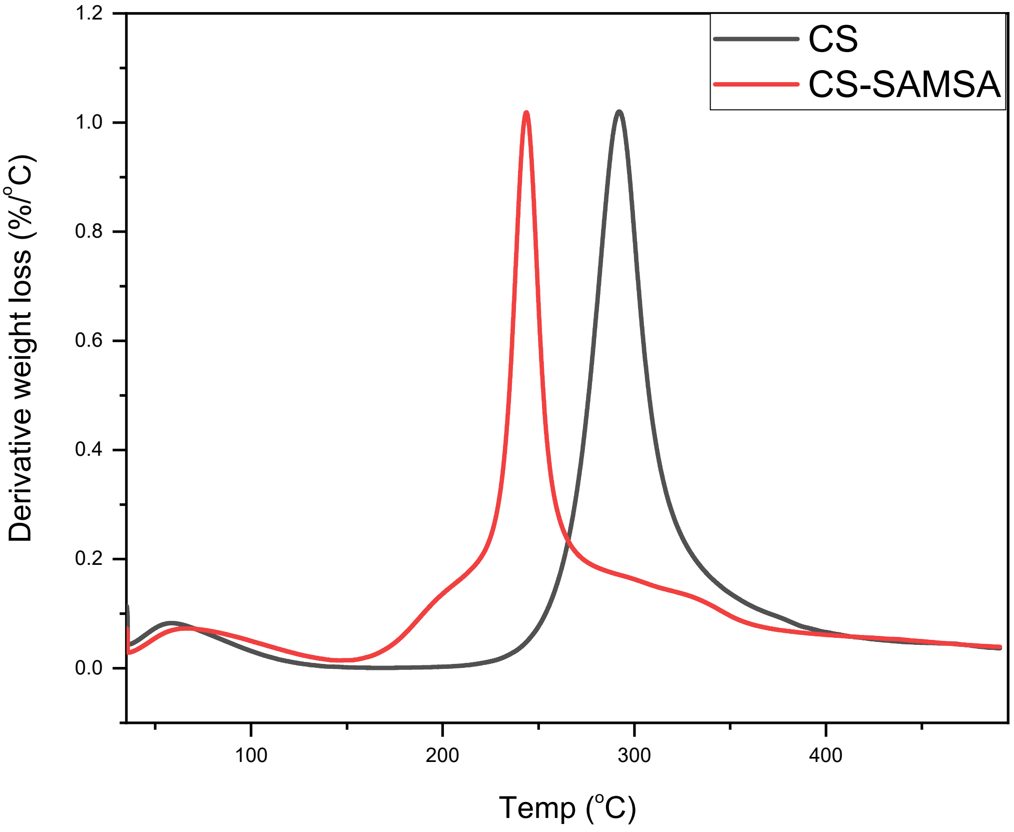

Figure S6: Differential Thermal Gravimetry (DTG) profile of CS and CS-SAMSA.

(a)

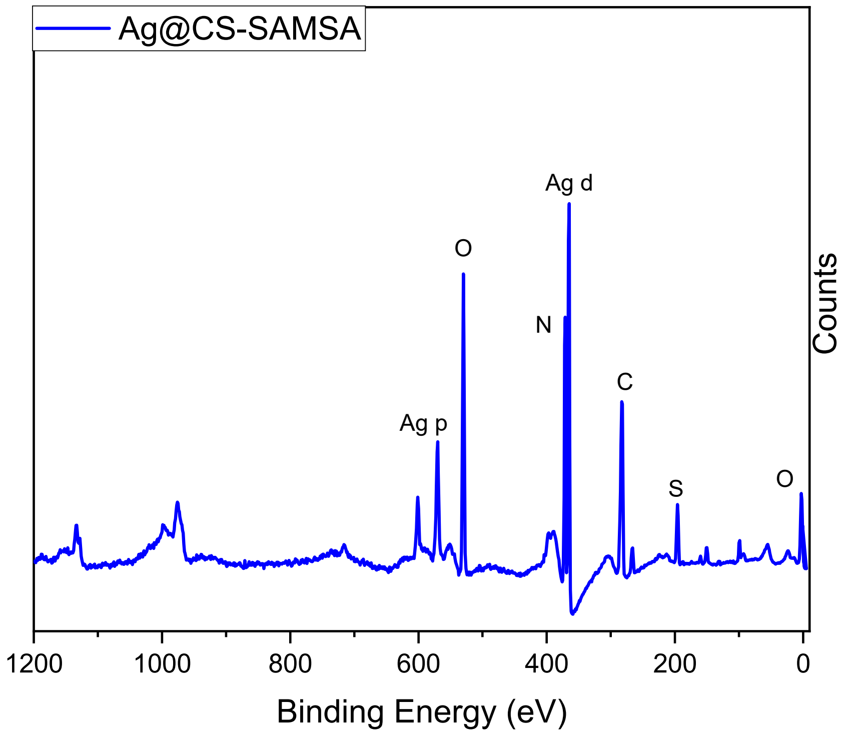
Ag@CS-SAMSA
Ag d
O
N
C
Ag p
S
O
Counts
1200
1000
800
600
400
200
0
Binding Energy (eV)

(b)

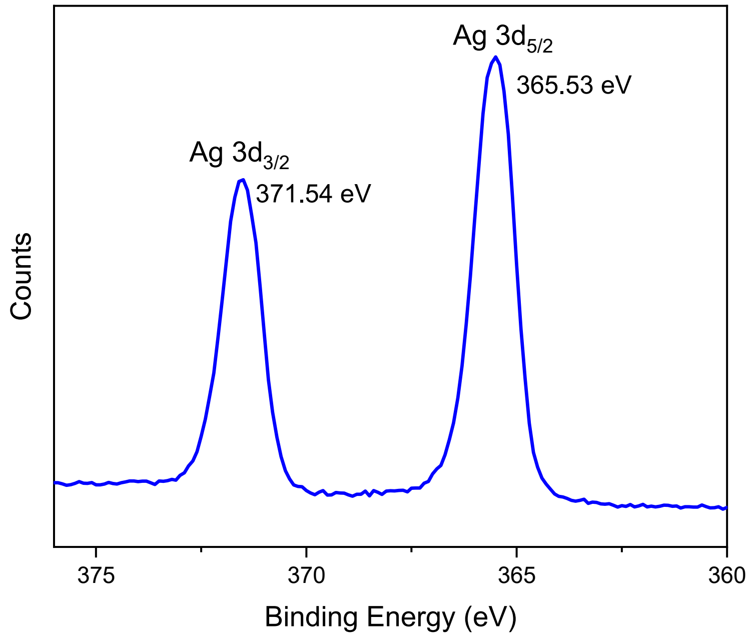
Ag 3d5/2
365.53 eV
Ag 3d3/2
371.54 eV
Counts
375
370
365
360
Binding Energy (eV)

(c)

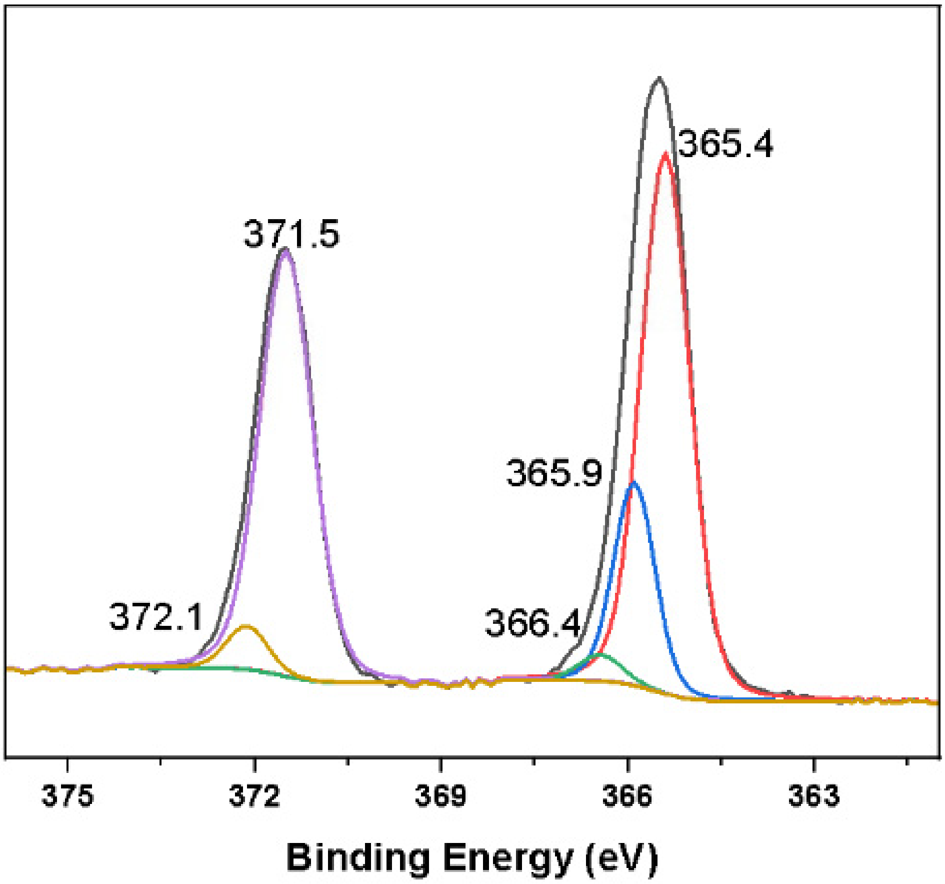

Figure S7: XPS profiles: (a) wide scan, (b) high resolution spectra, and (c) deconvoluted spectral bands.

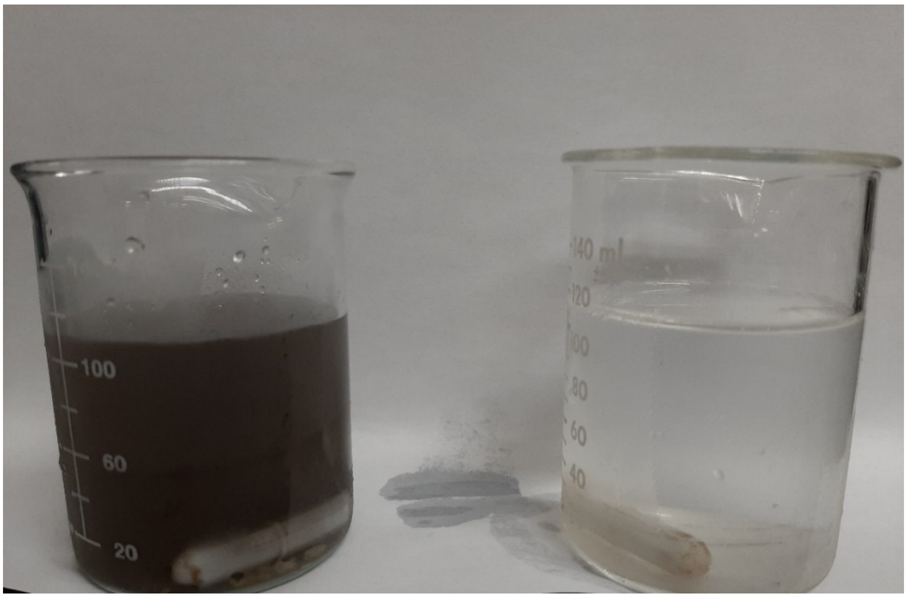

Ag@CS **Ag@CS-SAMSA**

Figure S8: Different appearance of Ag@CS-SAMSA (right) and Ag@CS (left) solution.

(a)

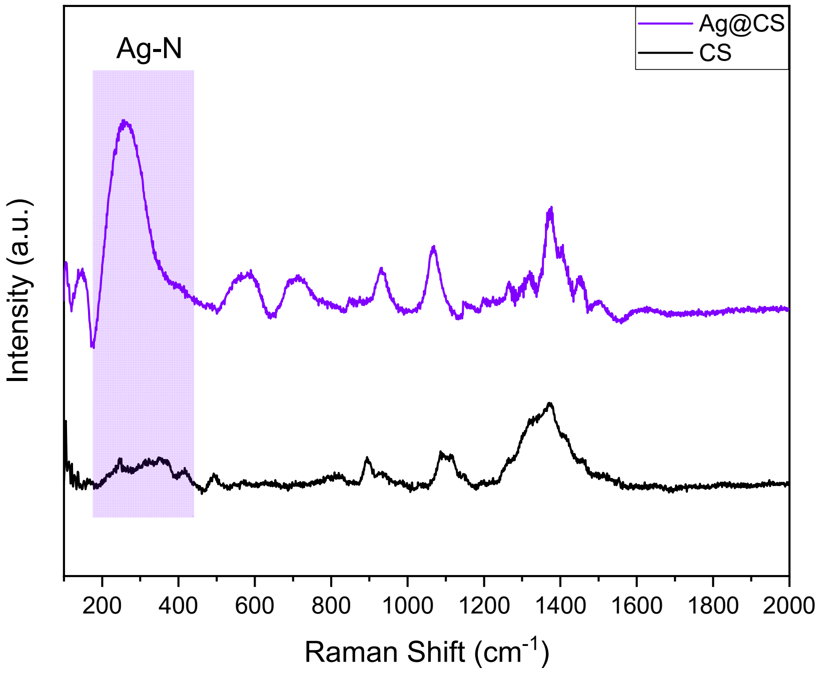

(b)

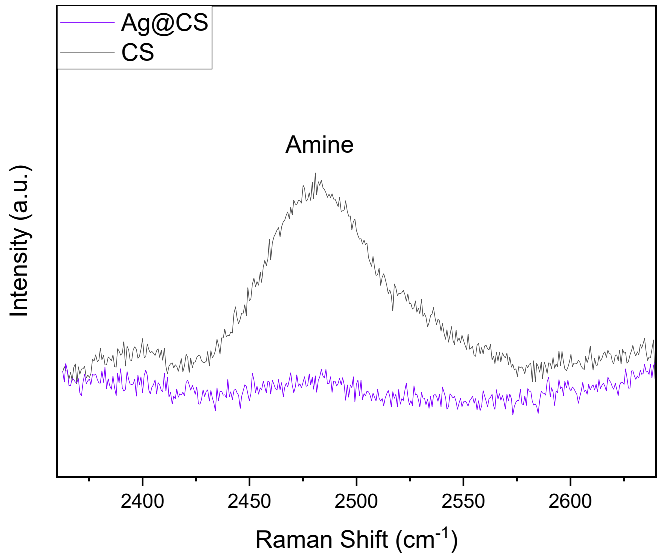

Figure S9: (a) FT-Raman spectra of CS (black) and Ag@CS (purple). The highlighted region shows the main difference after the addition of Ag NPs, and (b) FT-Raman spectra of CS (black) and Ag@CS (purple) for a static scan centered at 2550 cm^{-1}.

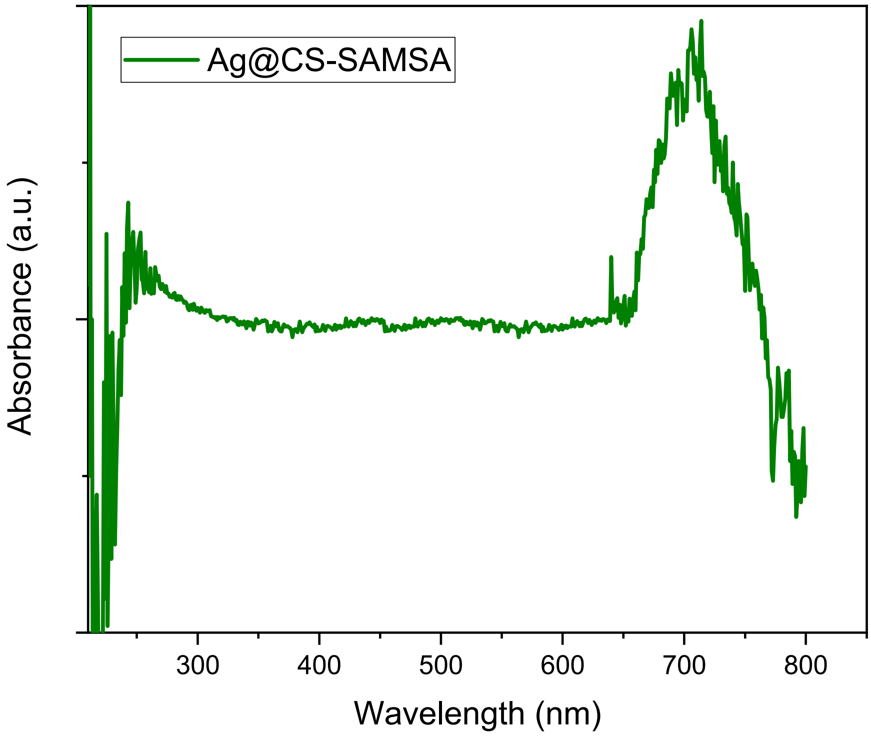

Figure S10: UV-Vis adsorption spectra of Ag@CS-SAMSA which shows the LSPR peak at 720 nm, close enough to the laser excitation wavelength of 785 nm.

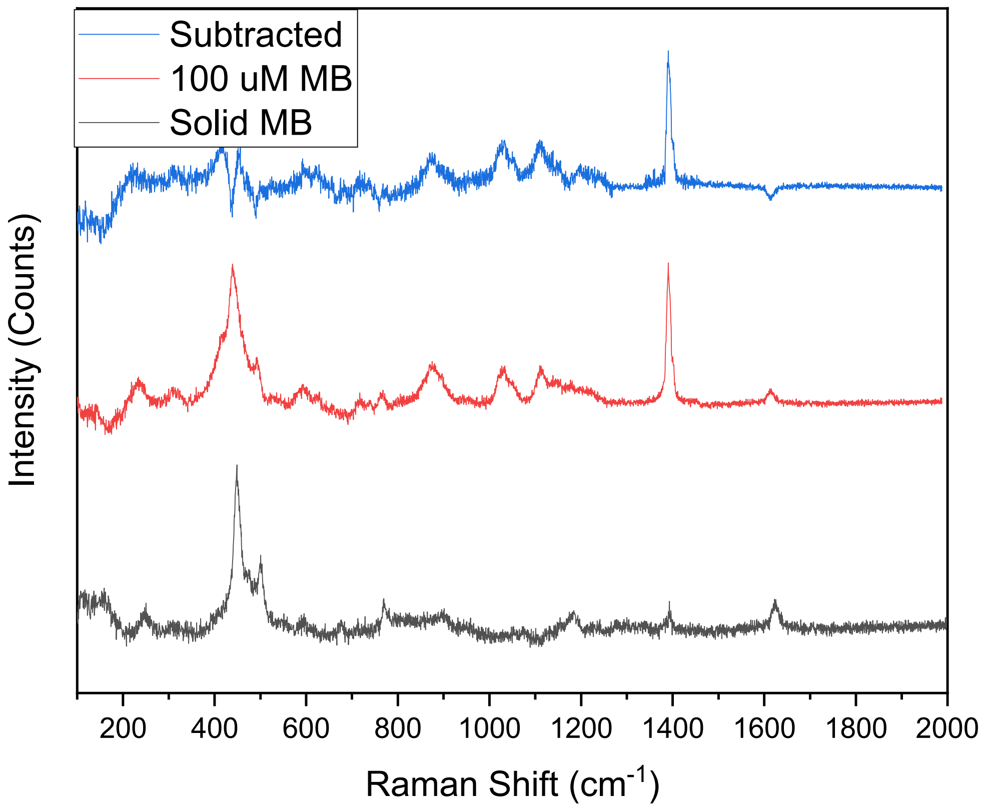

Figure S11: The black line is the subtracted Raman spectrum of Solid MB (black) and MB (10 µL, 100 µM) on Ag@CS-SAMSA (Red).

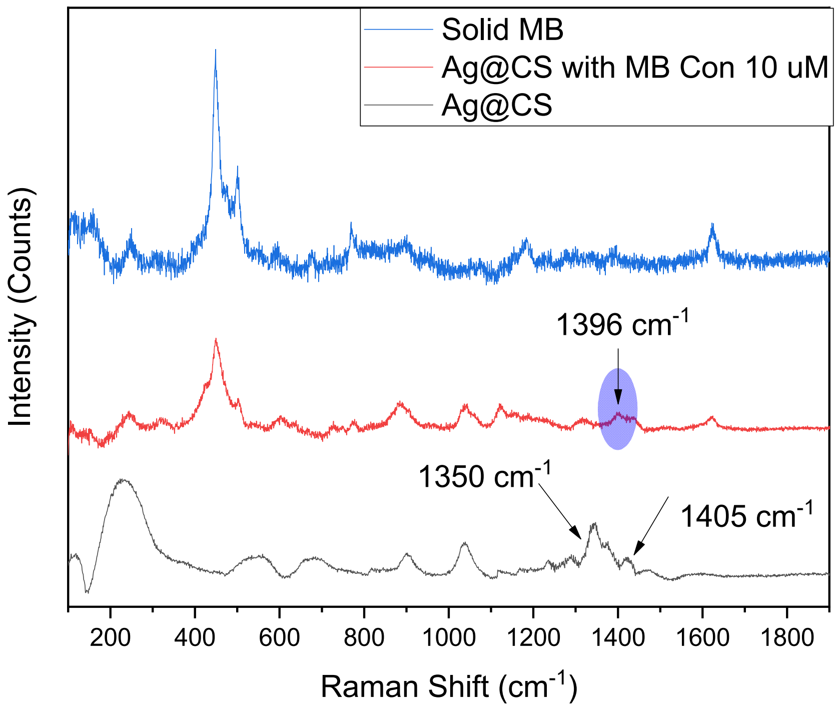

Figure S12: SERS spectra of MB (10 µL, 10 µM) onto the Ag@CS substrate.

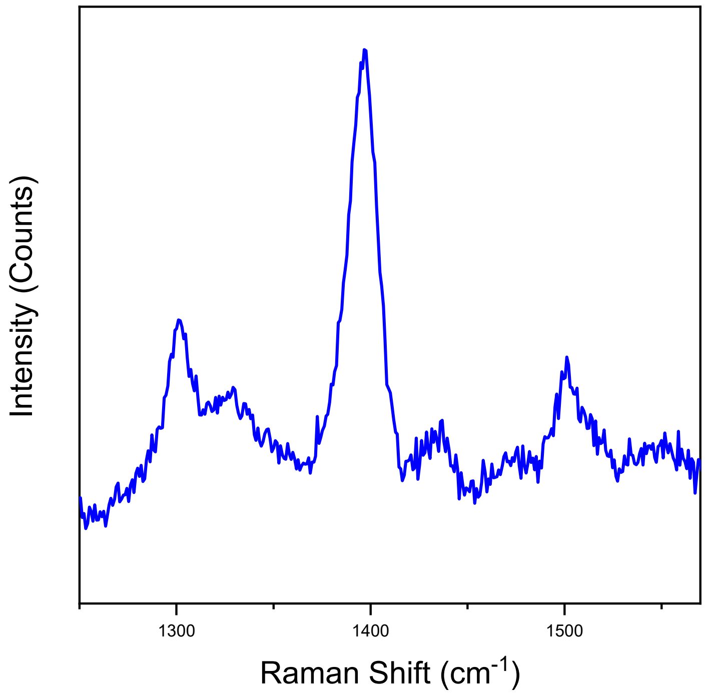

Figure S13: Raman spectrum of Ag@CS-SAMSA with MB (10 µL, 10 µL) Static scan centered at 1396 cm-1 (SERS peak).

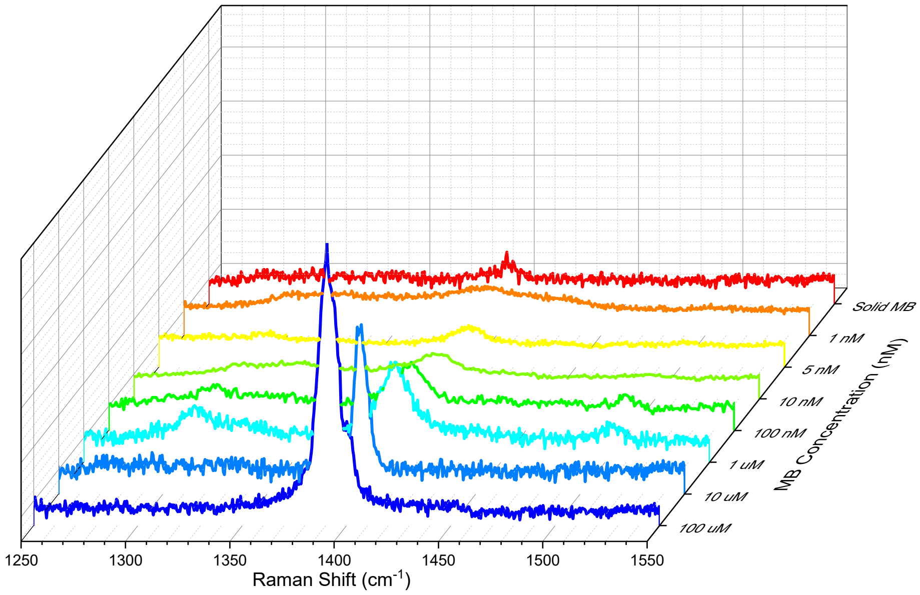

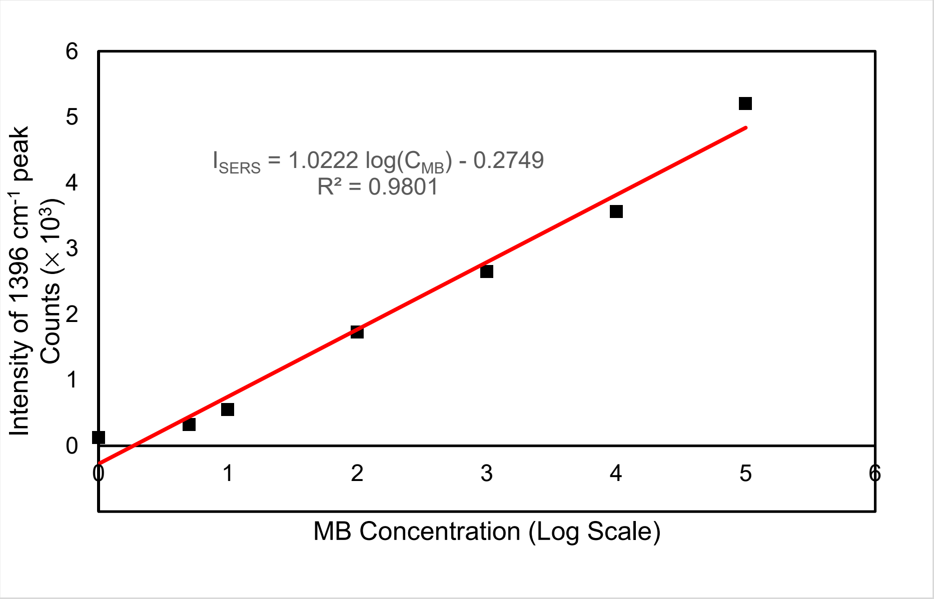

Figure S14: Top: Focus of SERS peak centered at 1396 cm^{-1} Bottom: calibration curve to calculate LOD and LOQ.

(a)

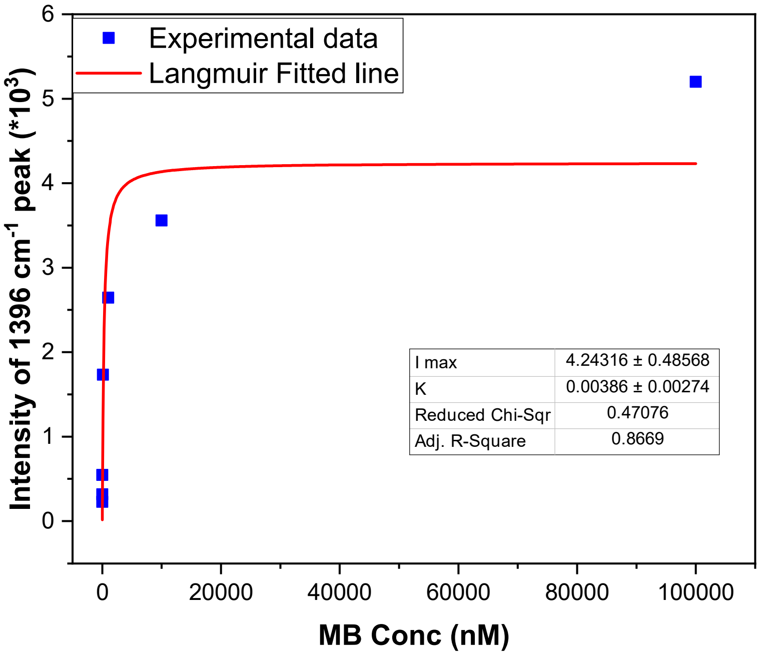

(b)

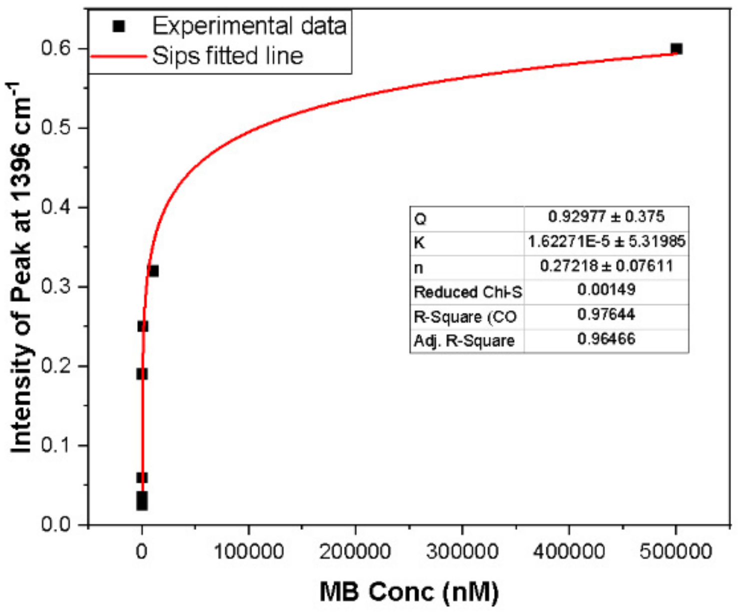

Figure S15: Adsorption isotherm of MB on SERS substrate fitted with (a) Langmuir and (b) Sips model. MB concentration: 1 nm to 100 µM, contact time 1 h at 23°C.

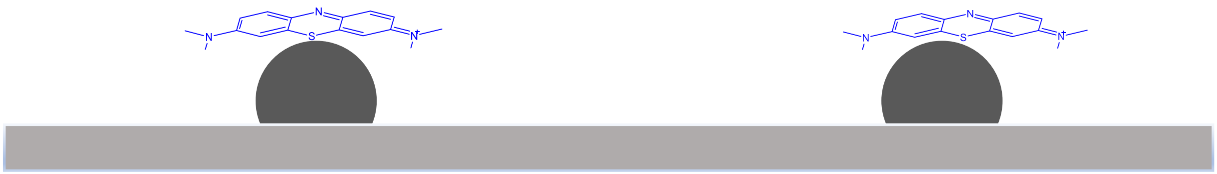

Figure S16: Schematic representation of adsorbed MB on Ag@CS-SAMSA to represent the binding geometry of MB with the substrate.

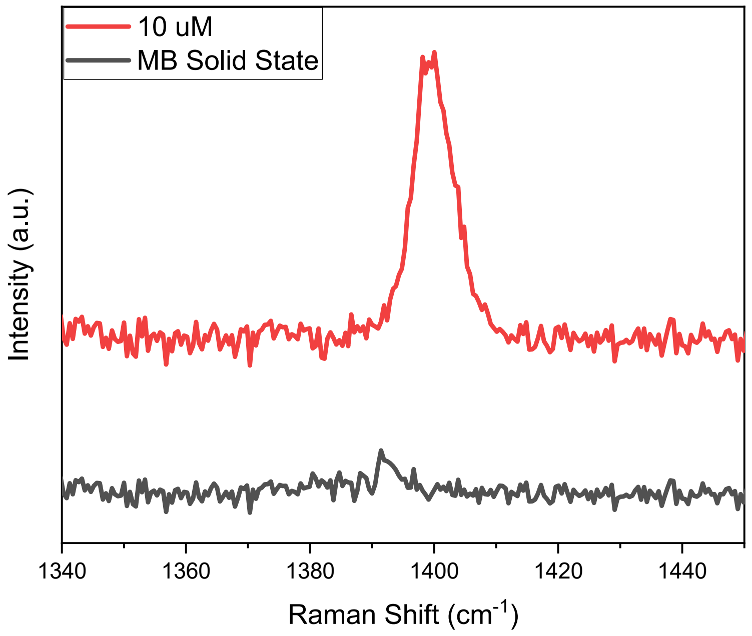

Figure S17: The Stokes Raman shift of the SERS band for substrate with MB (Red) and solid MB (Black). MB concentration (10 µM, 10 µL).

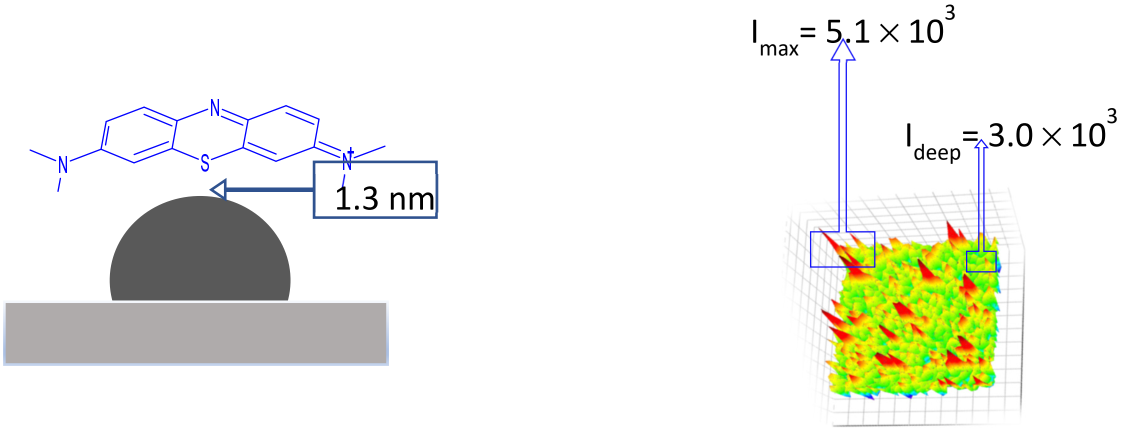

Figure S18: Schematic representation of adsorbed MB on Ag@CS-SAMSA to show the distance between MB and Ag NP adsorption site.

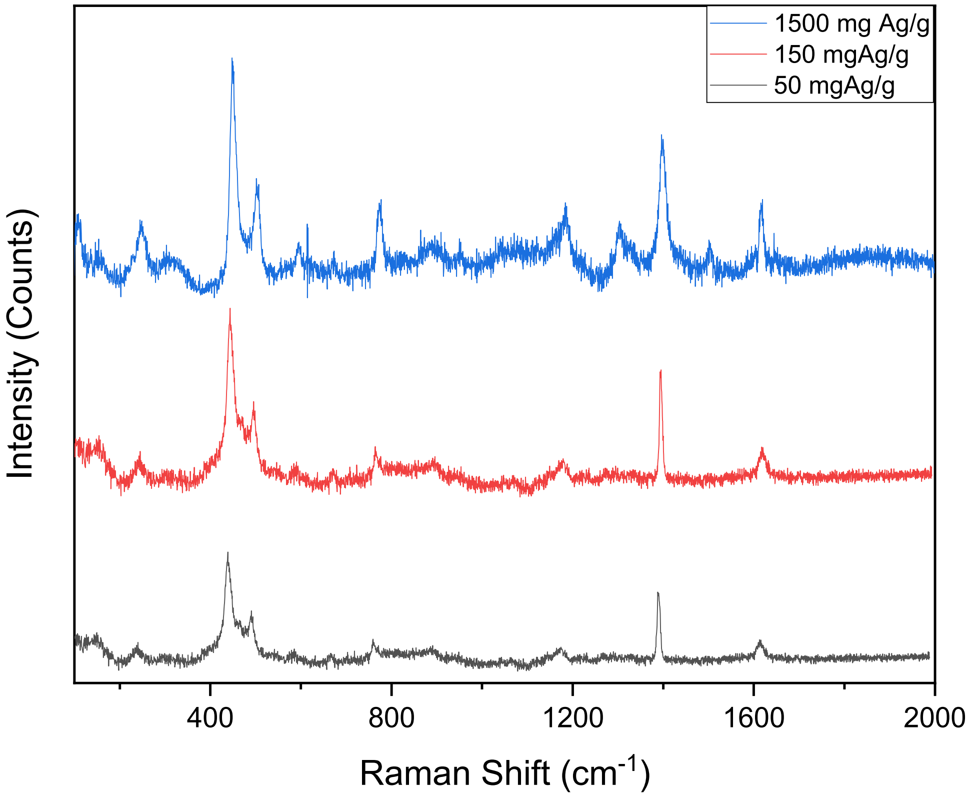

Figure S19: Effect of Ag NP concentration in the CS-SAMSA substrate. The concentration of $AgNO_3$ solution for 50 mg Ag/g of substrate is 50 mM, and for 1500 mg Ag/g of substrate is 150mM.

For SERS studies MB (10 µM, 10 µL) was dispersed on different substrate and dried at 23°C for 1 hour.

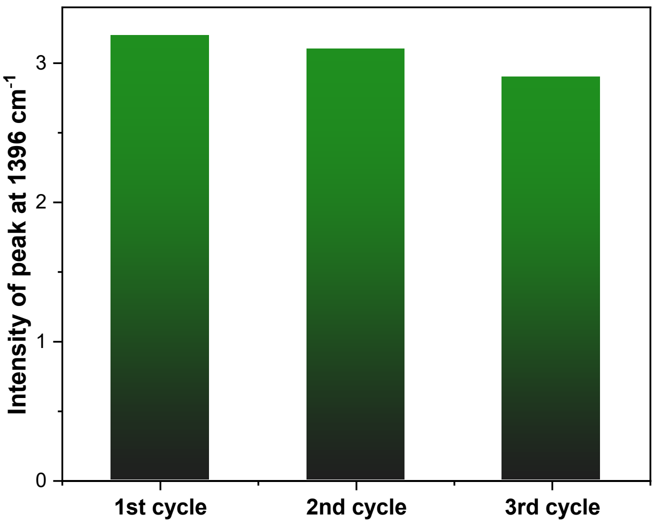

Figure S20: Regeneration cycles for Ag@CS-SAMSA loaded with MB. Adsorption cycle condition: MB concentration (10 µL, 10 µM) Desorption cycle condition: methanol and water washing

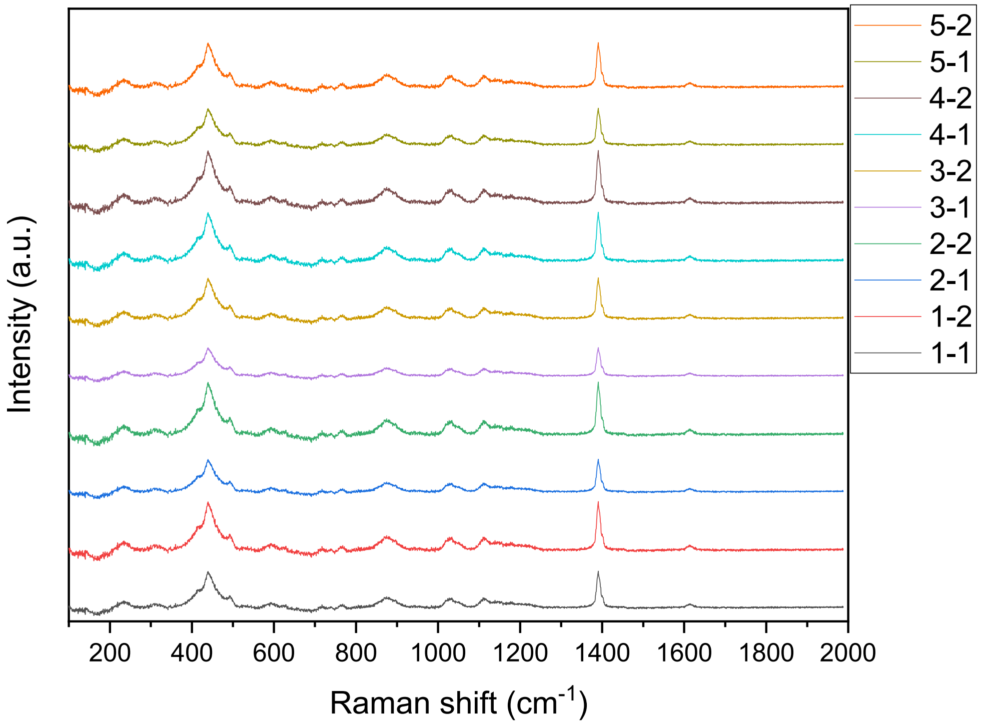

Figure S21: Reproducibility of the SERS substrate by MB (10 µL, 10 µM) for 2 selected sites of five different batches. The intensities are: 3.1, 3.2, 2.9, 3.0, 3.1, 3.4, 2.9, 3.1, 3.0, 2.9 ($\times10^3$ cps) and the RSD% is 5.2%.

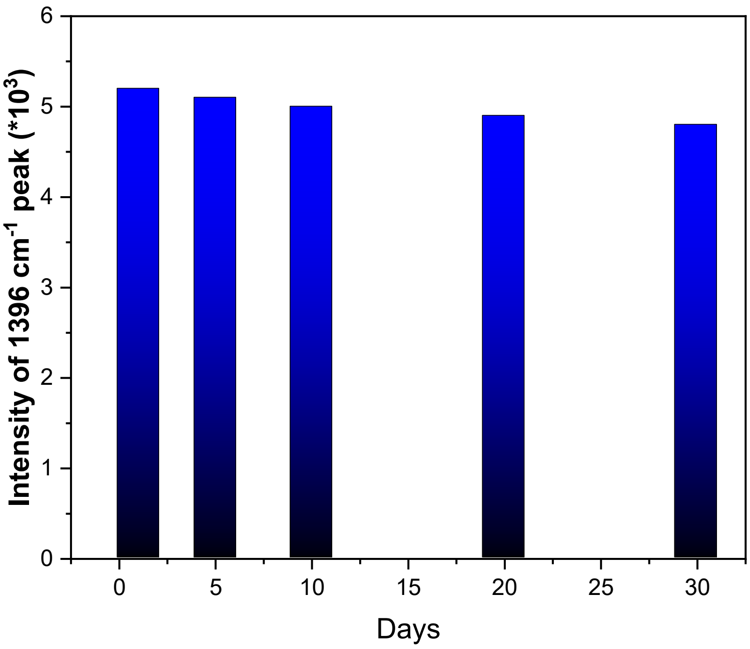

Figure S22: Storage Stability of substrate after 30days tested on 0, 5, 10, 20 and 30 days and the Raman scattering intensity was compared. MB (10 µL, 10 µM).

S6. Supplementary Tables

Table S1: The spectral positions and assignments of the Raman signatures.

Raman Peak	Description
226	Ag-N, Ag-S bending
440	OH Bending
490	CO-NH, C-C Bending
595	OH Bending
690	C-H Bending
773	C-H out of plane Bending
870	C-H Bending
890	C-C, C-N Stretching
920	C-C, C-H Stretching
1024	C-H Stretching
1058	C=O, C-O Stretching
1110	C-O, C-H Stretching
1183	Aromatic C-H Stretching
1312	C-O-C Stretching
1380	C-H Stretching
1390	Ring Stretching
1580	Graphene G-band
1620	C=N Stretching
1655	C=O Stretching
2450	Amine Stretching
2550	Thiol Stretching

Table S2: Langmuir and Sips best-fit adsorption parameters for MB with Ag@CS-SAMSA.

Langmuir			Sips			
I_{max}	K_s (nM^{-1})	Adj-R^2	I_{max}	K_s (nM^{-1})	n_s	Adj-R^2
4.24	3.86×10^{-3}	0.87	6.79	2.18×10^{-4}	0.35	0.95

Table S3: Comparison of EF and LOD of substrates from the literature, along with the results reported in this study.

SERS Substrate	Dye	EF	LOD	Ref
Silica core with a silver cap	MB	4.2×10^7	-	1
Graphene Foam Decorated with Ag NPs	MB	5.0×10^4	1 nM	2
Chitosan Nanofibers Functionalized with Ag NPs	RhoB	-	10^4 nM	3
Ag NPs-loaded chitosan foam(3D)	Rho6G	-	1 nM	4
Gelatin–nanogold bioconjugate	Rose Bengal	-	360 nM	5
WO_2/C ultrathin nanowire	Rho6G	1.3×10^6	10 nM	6
Poly(vinyl butyral-co-vinyl alcohol-co-vinyl acetate) (PVVV) with Ag NPs	Rho6G	~ 10^7	5.6 nM	7
**This Work**	**MB**	**2.6×10^8**	**1.6 nM**	-

Note: Rhodium 6G (Rho6G), Rhodium B (Rho B), and methylene blue (MB)

References:

1. Klotz, I.M.; Heiney, R.E. Introduction of Sulfhydryl Groups into Proteins Using Acetylmercaptosuccinic Anhydride. Arch Biochem Biophys 1962, 96, 605–612, doi:10.1016/0003-9861(62)90345-4.
2. Dolatkhah, A.; Wilson, L.D. Magnetite/Polymer Brush Nanocomposites with Switchable Uptake Behavior Toward Methylene Blue. ACS Appl Mater Interfaces 2016, 8, 5595–5607, doi:10.1021/acsami.5b11599.
3. Xue, C.; Wilson, L.D. Preparation and Characterization of Salicylic Acid Grafted Chitosan Electrospun Fibers. Carbohydr Polym 2022, 275, 118751, doi:10.1016/j.carbpol.2021.118751.
4. Chattopadhyay, D.P.; Inamdar, M.S. Aqueous Behaviour of Chitosan. Int J Polym Sci 2010, 2010, doi:10.1155/2010/939536.

5. Costa, C.N.; Teixeira, V.G.; Delpech, M.C.; Souza, J.V.S.; Costa, M.A.S. Viscometric Study of Chitosan Solutions in Acetic Acid/Sodium Acetate and Acetic Acid/Sodium Chloride. Carbohydr Polym 2015, 133, 245–250, doi:10.1016/j.carbpol.2015.06.094.

6. De Oliveira, A.M.; Franco, T.T.; Oliveira Junior, E.N. De Physicochemical Characterization of Thermally Treated Chitosans and Chitosans Obtained by Alkaline Deacetylation. Int J Polym Sci 2014, 2014, doi:10.1155/2014/853572.

7. Xue, C.; Wilson, L.D. A Structural Study of Self-Assembled Chitosan-Based Sponge Materials. Carbohydr Polym 2019, 206, 685–693, doi:10.1016/j.carbpol.2018.10.111.

8. Xiao, G.N.; Man, S.Q. Surface-Enhanced Raman Scattering of Methylene Blue Adsorbed on Cap-Shaped Silver Nanoparticles. Chem Phys Lett 2007, 447, 305–309, doi:10.1016/j.cplett.2007.09.045.

9. Srichan, C.; Ekpanyapong, M.; Horprathum, M.; Eiamchai, P.; Nuntawong, N.; Phokharatkul, D.; Danvirutai, P.; Bohez, E.; Wisitsoraat, A.; Tuantranont, A. Highly-Sensitive Surface-Enhanced Raman Spectroscopy (SERS)-Based Chemical Sensor Using 3D Graphene Foam Decorated with Silver Nanoparticles as SERS Substrate. Sci Rep 2016, 6, doi:10.1038/srep23733.

10. Prikhozhdenko, E.S.; Lengert, E. V.; Parakhonskiy, B. V.; Gorin, D.A.; Sukhorukov, G.B.; Yashchenok, A.M. Biocompatible Chitosan Nanofibers Functionalized with Silver Nanoparticles for Sers Based Detection. In Proceedings of the Acta Physica Polonica A; Polish Academy of Sciences, February 1 2016; Vol. 129, pp. 247–249.

11. Wang, C.; Wong, K.W.; Wang, Q.; Zhou, Y.; Tang, C.; Fan, M.; Mei, J.; Lau, W.M. Silver-Nanoparticles-Loaded Chitosan Foam as a Flexible SERS Substrate for Active Collecting Analytes from Both Solid Surface and Solution. Talanta 2019, 191, 241–247, doi:10.1016/J.TALANTA.2018.08.067.

12. Suarasan, S.; Focsan, M.; Maniu, D.; Astilean, S. Gelatin-Nanogold Bioconjugates as Effective Plasmonic Platforms for SERS Detection and Tagging. Colloids Surf B Biointerfaces 2013, 103, 475–481, doi:10.1016/j.colsurfb.2012.10.046.

13. A Highly Sensitive and Stable SERS Substrate Using Hybrid Tungsten Dioxide/Carbon Ultrathin Nanowire Beams. J Mater Chem C Mater 2018, 6, 3200–3205.

14. Hariprasad, E.; Radhakrishnan, T.P. In Situ Fabricated Polymer-Silver Nanocomposite Thin Film as an Inexpensive and Efficient Substrate for Surface-Enhanced Raman Scattering. Langmuir 2013, 29, 13050–13057, doi:10.1021/la402594j.
